# Enhancing Patient Care With the “David and Wendy” Approach: The Effectiveness of Ward Round Documentation Proformas

**DOI:** 10.7759/cureus.50143

**Published:** 2023-12-07

**Authors:** Georgia M Taylor, Brittany Long, John Nguyen, Nathan Brunott

**Affiliations:** 1 General Surgery, Cairns Base Hospital, Cairns, AUS

**Keywords:** surgery, general surgery, checklist, quality improvement, documentation

## Abstract

Background

Surgical ward round documentation, essential for high-quality patient care, is often completed poorly. The advent of electronic medical records offers an opportunity to introduce proformas, aiding junior staff in completing notes both timely and accurately. We aimed to assess whether the introduction of a proforma would improve the quality and speed of ward round documentation.

Methods

We completed a prospective cohort analysis of ward round documentation at a single institution. Analysis was conducted on the documentation of a single surgical team over a 10-week period, comprising five weeks of baseline data collection followed by five weeks with implementation of a proforma. This proforma was based on the "David & Wendy" acronym, encompassing diet, activity, vital signs, investigations/IV therapy, drains/lines, wound assessment, examination findings, nursing concerns, drugs/deep vein thrombosis (DVT) prophylaxis, and barriers to discharge.

Results

A total of 711 ward round notes were analyzed, 349 with proforma and 362 without. Statistically significant improvements were observed in the documentation of diet, activity, investigations/IV therapy, drains/lines, wound assessment, nursing concerns, drugs/DVT prophylaxis, and barriers to discharge (p < 0.05) with proforma use. No significant difference was noted in the documentation of vital signs or examination findings. The time taken to finalize ward round notes was significantly reduced with the proforma (M = 31.28 vs. 60.05 minutes, p < 0.001).

Conclusion

The introduction of the David & Wendy proforma significantly improved the speed and quality of documentation for key surgical ward round information during our study.

## Introduction

The daily ward round is a keystone in the modern surgical management of patients in hospitals, and its quality, or lack thereof, has been linked to higher rates of complications and worse outcomes for patients [1]. Contrary to popular belief, the majority of mortality in surgical patients is not a result of the surgery itself but rather from complications that arise on the wards and a failure to rescue these patients [2]. Poor documentation not only negatively impacts patient care but also affects the functionality of other teams, jeopardizes billing, and places clinicians and the hospital at significant medico-legal risk [1,3]. Surgical ward rounds are often a setting for poor documentation [4]. They are often short; a New Zealand study found that the average time spent at a patient's bedside was just 2 minutes and 17 seconds [4]. During this brief time, a multitude of information is reviewed and collated, including charts, investigations, verbal reports, and examination findings [1]. Finally, junior doctors with variable medical and surgical experience often complete the documentation, leading to errors [5]. The combination of these factors has, therefore, led to very poor documentation of the surgical ward round [1,5] and exemplifies the need to develop systems to improve this. 

Surgery has long utilized checklists to enhance patient care, with the World Health Organisation surgical safety checklist being a prime example of how checklists can revolutionize patient care [6]. Surgical ward round proformas, documents outlining minimum standards for documentation and providing prompts for key issues, have been studied for their ability to improve documentation quality and staff value [7-10]. Techniques like stickers [7-9] and checklists [7,10] have proven effective. A 2021 systematic review found that while the majority of studies (88%, 8 out of 9) reported improvements in surgical documentation with checklist usage, they still noted a lack of high-level evidence supporting their use [11]. Additionally, research regarding the use of electronic medical records and the development of proformas to enhance documentation is limited.

**Figure 1:**
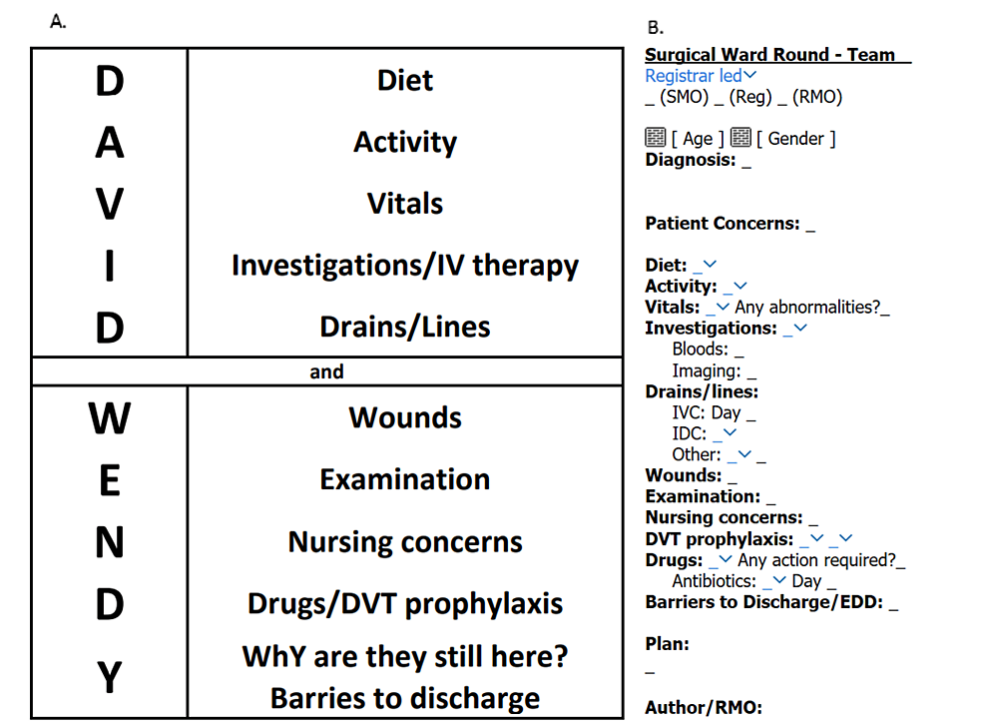
A: Explanation of David and Wendy. B: Example of unfilled ward round proforma. Underscores allow for fast movement between sections. Dropdown menus have prefilled responses for common outcomes specific to our institution and local policies (e.g., Enoxaparin for DVT prophylaxis), which may or may not be utilized depending on preference (see Appendix 1 for a full list of dropdowns). DVT: Deep vein thrombosis; SMO: Senior medical officer; Reg: Registrar; RMO: Resident medical officer; IVC: Intravenous cannula; IDC: Indwelling catheter; EDD: Estimated date of discharge.

The use of memorable acronyms as cognitive tools has been successfully implemented in various medical specialties to reduce errors and standardize practice where solid guidelines exist [12]. For example, the FAST HUG acronym (Feeding, Analgesia, Sedation, Thromboprophylaxis, Head of bed elevated, Ulcer prophylaxis, Glucose control) was developed as an ICU checklist to minimize common complications such as malnutrition and venous thromboembolism. It encompasses aspects that can often be neglected when the focus is primarily on medical diagnosis and advancing treatment [12]. The effectiveness of this approach is emphasized by the need for the checklist to be concise and the acronym memorable [12]. Its uptake and success cannot be understated. It has been shown to decrease ventilator-associated pneumonia, decrease mortality, and increase nursing staff's understanding of medical plans, and is now in widespread use [13-15]. We aimed to develop an acronym-based proforma within ieMR (our electronic medical record) to enhance the quality, accuracy, and utility of surgical ward round documentation without compromising time efficiency. We present David & Wendy (Diet, Activity, Vitals, Investigations/IV therapy, Drains, Wounds, Examination, Nursing concerns, Drugs/DVT prophylaxis, and Y - why are they still in the hospital - or barriers to discharge) as our solution (Figure 1).

## Materials and methods

This quality assurance study was conducted at Cairns Hospital. An ethics exemption was granted by the Far North Queensland Human Research Ethics Committee (FNQ HREC Reference: Project EX/2022/QCH/90904). Over a 10-week period (November 2022 to January 2023), ward round notes from the electronic medical record (iEMR) of a surgical team at Cairns Hospital were reviewed. Documentation of the listed parameters was recorded and stored on a secure, password-protected MS Excel™ spreadsheet within the Queensland Health Computer network. No patient demographics or clinical details were included in the study; the data was de-identified and subsequently tabulated. Documentation by a single surgical team was carried out in a standard fashion for five weeks without any intervention; this was then largely up to the interns completing the note and the registrar guiding the ward round as to what was or was not included. Subsequently, a ward round proforma was introduced and utilized for an additional five-week period. The proforma (Figure 1B) provided several headings to prompt their discussion and documentation. It included drop-down menus for rapid completion of common responses (e.g., Enoxaparin for DVT prophylaxis) but also permitted free text entries. The team also had the option to ignore the proforma and write entirely their own notes above or below. Data from the ward round notes over the 10-week period were categorized based on the use or non-use of the proforma and analyzed for the outcomes described.

The study population comprised all patients admitted under the surgical team during the 10-week period. There were no exclusion criteria, and patients may or may not have undergone surgery. Patient demographics and information related to diagnosis, interventions, and outcomes were not collected or analyzed, as these were deemed unrelated to the assessed outcomes. Given the data collection timeframe, it was assumed that these characteristics would be evenly distributed between the two groups, consistent with the nature of an acute surgical unit. The team admitted a range of acute and elective surgical pathologies in a range of general surgical subspecialties; however, the consultants had a predominance for breast, hernia, and hepatobiliary surgeries in their elective work. Ward round notes were categorized as either 'proforma used' or 'proforma not used,' and nominal data were collected on whether the notes specifically mentioned diagnosis, diet, mobility status, vital signs, investigations/IV therapy, drains/lines, wound assessment, examination findings, nursing concerns, drugs/DVT prophylaxis, and barriers to discharge. The start and completion times of the ward round notes (in our electronic record, completion is indicated by the signing of the note, which may or may not occur during the ward round) were also recorded. Where appropriate, summary statistics are presented as number (%) for binary and categorical data, and mean (SD) for normally distributed continuous data. Variables were compared using Fisher's exact test. Paired sample t-tests were performed to compare the mean time taken to finalize ward round documentation between the two groups, and effect sizes were determined using the Cohen d method. Stata (version 15.0) was used throughout, and the level of significance was set at p < 0.05.

## Results

**Figure 2:**
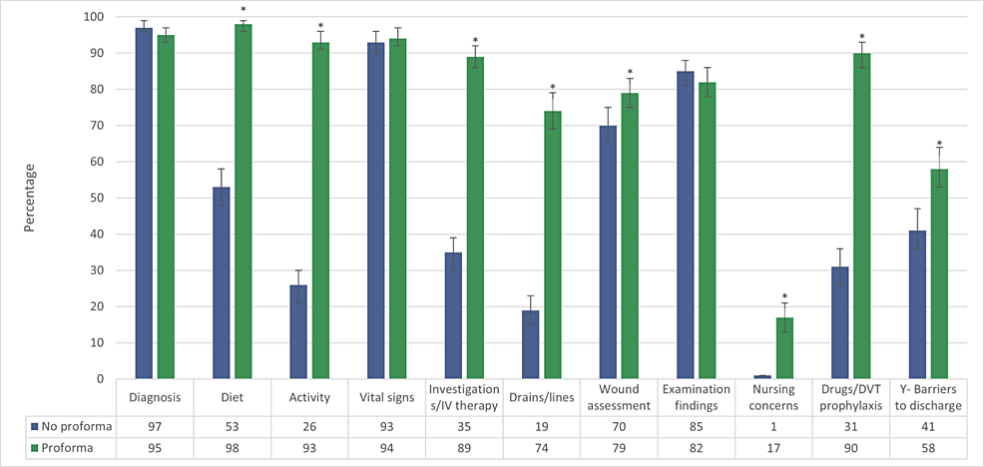
Total % of the ward round notes with documentation of the parameters assessed between the two groups. * denotes statistical significance (p < 0.05).

A total of 711 ward round notes were analyzed, 349 where the proforma was used and 362 where the proforma was not used. Documentation of the ward round note parameters for the two groups is presented in Figure 2. There was a statistically significant improvement in documentation of diet status (p = <0.001), mobility status (p = <0.001), investigations/IV therapy (p = <0.001), drains/lines (p = <0.001), wound assessment (p = 0.01), nursing concerns (p = <0.001), drugs/DVT prophylaxis (p = <0.001), and barriers to discharge (p = <0.001) when the proforma was used. There was no statistically significant difference in documentation of diagnosis (p = 0.12), vital signs (p = 0.54), or examination findings (p = 0.37) between groups. A comparison of these two groups is demonstrated in Figure 2.

**Figure 3:**
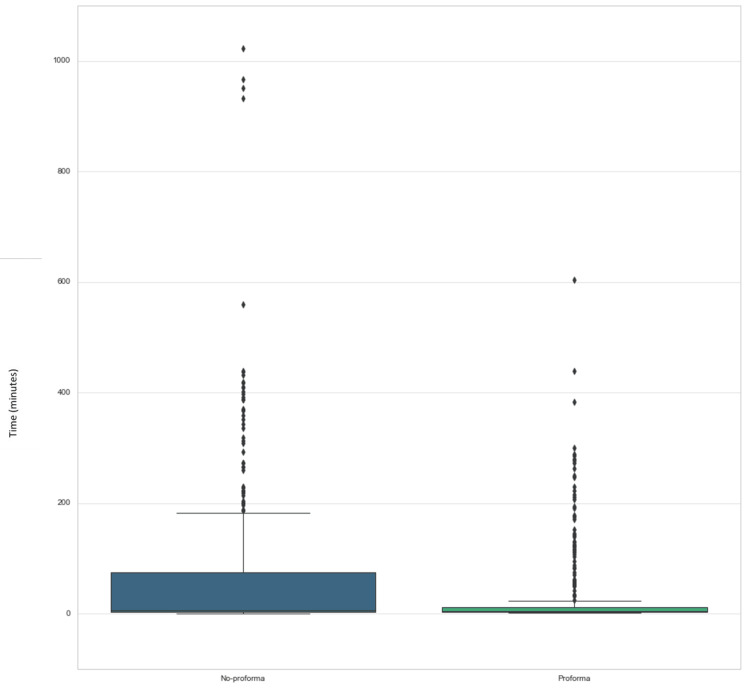
Box and whisker plot of time taken to completion of notes. Significantly less time to notes completion when the proforma was used (p < 0.01), with less variance in the data.

There was a significant decrease in mean time taken to finalize ward round notes per patient (M = 31.28 minutes, SD 72.01 vs. M = 60.05 minutes, SD 107.39); despite significant outliers, the median was also reduced (5 minutes for no proforma, 6 minutes for proforma) (Figure 3). 

## Discussion

The introduction of a surgical ward round proforma in our study led to significantly increased documentation rates of key elements of surgical care. These elements include diet and mobility status, investigations and IV therapy, drains and lines, wound assessment, nursing concerns, drugs and DVT prophylaxis, and barriers to discharge. Diet is a crucial aspect of a surgical ward round for two main reasons. Firstly, post-operative or acutely admitted general surgical patients often have initially restricted diets, and up-titration of this is a progressive step to discharge. Secondly, there is a known association between malnutrition and poorer outcomes in critically ill patients [16]. Prior to implementing the proforma, dietary status documentation was observed to be inadequate, contributing significantly to the workload and communication challenges between nurses and junior doctors. This was corroborated by the research by Al-Mahrouqi H et al. (2013) [7] and their attempt at a proforma; however, in contradiction to their study, we had very promising improvements in dietary records (53% pre-proforma, and 98% with-proforma, p < 0.001). Mobility status was likewise included to improve clarity and decrease team communication workload. The usefulness of documentation of some areas, specifically of DVT prophylaxis and review of medications, has two fulcrums of action. The first is the clear documentation of plans for communication between groups. The second purpose is to act as a reminder of these potentially life-saving interventions, which are essential for all patients, to prevent oversight, such as the cessation of antibiotics for improved antibiotic stewardship. In this regard, our proforma was designed with similarities to the FAST HUG approach [12] used in ICU, which has been associated with reduced mortality [14]. Despite the extensive evidence supporting the systematic use of thromboprophylaxis and its role in reducing mortality [17], its documentation in our study before the implementation of the proforma was notably low (31%). This documentation significantly improved with the use of the proforma (90%, p < 0.001).

A major concern regarding the introduction of increased documentation was the possible increase in time taken; however, our study demonstrated that the use of the proforma decreased the time taken to finalize the notes. This is unique to our research and is likely an effect of electronic medical records, where notes can be completed at the time of the ward round but not finalized for several hours. However, the shorter time to finalization is still significant and may relate to increased discussion at the time of the ward round and a decrease in the need to seek clarification during the day prior to finalizing the notes. 

Key to the development of our proforma was the creation of an easy-to-remember mnemonic - David & Wendy. This mnemonic extends beyond the completion of the ward round and the use of the iEMR proforma; it serves as a mental checklist for reviewing any surgical patient on the ward or, likewise, for the initial post-operative plan in an operation report. While junior doctors may find the use of the proforma important for their day and for communication, more senior doctors may use this subliminally as a memory device to ensure they are attending to common problems for surgical patients, such as drains and their decision for removal. Not every element of the mnemonic needs to be documented for every patient at every review, but it provides a comprehensive guide to ensure no critical aspect is overlooked.

Strengths and weaknesses 

Our study included a robust number of patients and charts, with roughly equal groups, providing high statistical power. Using the same team and junior doctors helped control for variation in skill and quality. However, since the proforma was introduced in the second half of their first term as surgical interns, some of the measured effects might be attributed to increasing knowledge and competence during this period. Maintaining the same team also standardized differences in patient cohorts and team demands, but patient demographics and complexity were not assessed. The junior doctors were blinded to the pre-proforma notes collection; however, blinding post-introduction was not feasible, potentially introducing bias due to the Hawthorne effect (modification of behavior in response to being observed)[18]. Our study did not account for carrying errors, and since the content of the notes was not scrutinized, we cannot comment on their accuracy.

Further study 

Previous qualitative reviews of ward round documentation have indicated mixed perceived benefits for nursing staff [7,15,19], with its usefulness to medical staff remaining untested. Staff buy-in across medical, nursing, and allied health streams is crucial for long-term adoption and represents an important area for future study. During our trial, we observed that nursing staff tend to be more present to voice their concerns during the ward rounds. Their inclusion in the round allows the nursing team to be part of the review and management, which could be quantified with further study. Another potential research focus is quantifying the impact on junior doctors' workload and the number of phone calls received. Anecdotal feedback suggested that the proforma used in this study was somewhat complex and challenging to extract information from, highlighting a clear distinction between the needs of medical and nursing staff from ward round notes. However, it was generally considered favorable, especially by nursing and allied health staff. Further, Tailoring the format to better suit individual units may be necessary. Measurement of prolonged, widespread use and its effect on key patient-centered outcomes would promote the use of the proforma, for example, charting of DVT prophylaxis, length of stay b

## Conclusions

 

The introduction of a ward round proforma based on the David and Wendy acronym can significantly enhance documentation in areas critical to inter-departmental communication and patient-centered outcomes. Our research demonstrated statistically significant improvements in documenting diet, activity, investigations/IV therapy, drains/lines, wound assessment, nursing concerns, drugs/DVT prophylaxis, and barriers to discharge. We also observed a reduction in the time taken to finalize notes. While not every item on this list may be relevant for all patients, nor is it an exhaustive list of all essential aspects of surgical patient care and subspecialties, it serves as a guideline to help both junior and senior staff avoid common pitfalls in the fast-paced surgical environment.

## Appendices

Dropdown options utilised.

**Table 1:** Dropdown options utilised

Section	Dropdown options
Registrar led	Registrar led (prefilled)
	Consultant led
Diet	Full
	Free fluids
	Clear fluids
	NBM
	Low residue
	Low fat
Activity	Independent
	Awaiting physio review
	Below baseline
	Bed bound
	Limited by pain
Investigations	Reviewed
	Awaiting
	Not required
Drains/Lines: IDC	Yes – Day _
	No
Drains/Lines: Other	Surgical drain
	N/A
	ICC
	Central line
	Arterial line
	PICC
DVT prophylaxis	Mechanical
DVT prophylaxis	Chemical – charted
	Chemical – withhold
	Chemical - contraindicated
Drugs	reviewed
Antibiotics	No
	Piptaz
	Cef + met
	Triples
	Yes - _
	Augmentin IV
	Augmentin oral

Abbreviations - NBM = nil by mouth, IDC = indwelling catheter, N/A = not applicable, ICC = intercostal catheter, PICC = peripherally inserted central catheter, DVT = deep vein thrombosis, Piptaz = piperacillin and tazobactam, Cef + met = ceftriaxone and metronidazole, triples = amoxicillin, gentamicin and metronidazole, IV = intravenous

## References

[1] Pucher PH, Aggarwal R, Darzi A (2014). Surgical ward round quality and impact on variable patient outcomes. Ann Surg.

[2] Ghaferi AA, Birkmeyer JD, Dimick JB (2009). Variation in hospital mortality associated with inpatient surgery. N Engl J Med.

[3] Kightlinger R (1999). Sloppy records--the kiss of death for a malpractice defense. Med Ec.

[4] Creamer GL, Dahl A, Perumal D, Tan G, Koea JB (2010). Anatomy of the ward round: the time spent in different activities. ANZ J Surg.

[5] Fernando KJ, Siriwardena AK (2001). Standards of documentation of the surgeon-patient consultation in current surgical practice. Br J Surg.

[6] Haynes AB, Weiser TG, Berry WR (2009). A surgical safety checklist to reduce morbidity and mortality in a global population. N Engl J Med.

[7] Al-Mahrouqi H, Oumer R, Tapper R, Roberts R (2013). Post-acute surgical ward round proforma improves documentation. BMJ Qual Improv Rep.

[8] Alamri Y, Frizelle F, Al-Mahrouqi H, Eglinton T, Roberts R (2016). Surgical ward round checklist: does it improve medical documentation? A clinical review of Christchurch general surgical notes. ANZ J Surg.

[9] Krishnamohan N, Maitra I, Shetty VD (2019). The surgical ward round checklist: improving patient safety and clinical documentation. J Multidiscip Healthc.

[10] Tranter-Entwistle I, Best K, Ianev R, Beresford T, McCombie A, Laws P (2020). Introduction and validation of a surgical ward round checklist to improve surgical ward round performance in a tertiary vascular service. ANZ J Surg.

[11] Treloar EC, Ting YY, Kovoor JG, Ey JD, Reid JL, Maddern GJ (2022). Can checklists solve our ward round woes? A systematic review. World J Surg.

[12] Vincent JL (2005). Give your patient a fast hug (at least) once a day. Crit Care Med.

[13] Papadimos TJ, Hensley SJ, Duggan JM (2008). Implementation of the "FASTHUG" concept decreases the incidence of ventilator-associated pneumonia in a surgical intensive care unit. Patient Saf Surg.

[14] Ferreira CR, de Souza DF, Cunha TM, Tavares M, Reis SS, Pedroso RS, Röder DV (2016). The effectiveness of a bundle in the prevention of ventilator-associated pneumonia. Braz J Infect Dis.

[15] Karalapillai D, Baldwin I, Dunnachie G (2013). Improving communication of the daily care plan in a teaching hospital intensive care unit. Crit Care Resus.

[16] Giner M, Laviano A, Meguid MM, Gleason JR (19961). In 1995 a correlation between malnutrition and poor outcome in critically ill patients still exists. Nutrition.

[17] Gould MK, Garcia DA, Wren SM, Karanicolas PJ, Arcelus JI, Heit JA, Samama CM (2012). Prevention of VTE in nonorthopedic surgical patients: Antithrombotic Therapy and Prevention of Thrombosis, 9th ed: American College of Chest Physicians Evidence-Based Clinical Practice Guidelines. Chest.

[18] McCarney R, Warner J, Iliffe S, van Haselen R, Griffin M, Fisher P (2007). The Hawthorne effect: a randomised, controlled trial. BMC Med Res Methodol.

[19] Ng J, Abdelhadi A, Waterland P (2018). Do ward round stickers improve surgical ward round? A quality improvement project in a high-volume general surgery department. BMJ Open Qual.

